# Percutaneous Ablation of T1 Renal Masses: Comparative Local Control and Complications after Radiofrequency and Cryoablation

**DOI:** 10.3390/diagnostics13193059

**Published:** 2023-09-26

**Authors:** Lorenzo Bertolotti, Federica Segato, Francesco Pagnini, Sebastiano Buti, Andrea Casarin, Antonio Celia, Francesco Ziglioli, Umberto Maestroni, Giuseppe Pedrazzi, Velio Ascenti, Chiara Martini, Calogero Cicero, Massimo De Filippo

**Affiliations:** 1 Section of Radiology, Department of Medicine and Surgery, University of Parma, Maggiore Hospital, Via Gramsci 14, 43126 Parma, PR, Italy; lorenzo.bertolotti@unipr.it (L.B.); f.pagnini90@gmail.com (F.P.); massimo.defilippo@unipr.it (M.D.F.); 2 G.B. Rossi University Hospital, University of Verona, 37134 Verona, VR, Italy; federica.segato@studenti.univr.it; 3 Department of Medicine and Surgery, University of Parma–Oncology Unit, University Hospital of Parma, Via Gramsci 14, 43126 Parma, PR, Italy; sebastiano.buti@unipr.it; 4 Department of Radiology, San Bassiano Hospital, 36061 Bassano del Grappa, VI, Italy; andrea.casarin@aulss7.veneto.it (A.C.); calogero.cicero@aulss7.veneto.it (C.C.); 5 Department of Urology, San Bassiano Hospital, 36061 Bassano del Grappa, VI, Italy; antonio.celia@aulss7.veneto.it; 6 Department of Urology, Parma University Hospital, Via Gramsci 14, 43126 Parma, PR, Italy; fziglioli@ao.pr.it (F.Z.); umaestroni@ao.pr.it (U.M.); 7 Centre of Statistic, Department of Medicine and Surgery, University of Parma, 43126 Parma, PR, Italy; giuseppe.pedrazzi@unipr.it; 8 Postgraduate School of Radiodiagnostics, Policlinico Universitario, University of Milan, 20133 Milano, MI, Italy; velio.ascenti@unimi.it; 9 Department of Medicine and Surgery, University of Parma, Maggiore Hospital, Via Gramsci 14, 43126 Parma, PR, Italy

**Keywords:** radiofrequency ablation, cryoablation, renal mass, kidney cancer, thermal ablation, percutaneous ablation

## Abstract

The efficacy and complication rates of percutaneous radiofrequency ablation (RFA) and cryoablation (CA) in the treatment of T1 renal masses in two Northern Italy hospitals were retrospectively investigated. Eighty-two patients with 80 T1a tumors and 10 T1b tumors treated with thermal ablation from 2015 through 2020 were included. A total of 43 tumors in 38 patients were treated with RFA (2.3 ± 0.9 cm), and 47 tumors in 44 patients were treated with CA (2.1 ± 0.8 cm). The mean follow-up observation period was 26 ± 19 months. The major complications and efficacy, as measured using the technical success and local tumor recurrence rates, were recorded. There were three (6.9%) technical failures with RFA and one (2.1%) with cryoablation (*p* = 0.30). Among the 40 tumors that were successfully treated with RFA, 1 tumor (2.5%) developed local tumor recurrence; 5/46 tumors that were treated with cryoablation (10.8%) developed local tumor recurrence (*p* = 0.17). T1b lesions (4.0 ± 0.7 cm) resulted in 1/6 technically unsuccessful cases with RFA and 0/4 with CA. No recurrent disease was detected in the T1b lesions. Major complications occurred after 2.3% (1/43) of RFAs and 0/47 of cryoablation procedures. RFA and cryoablation are both effective in the treatment of renal masses. Major complications with either procedure are uncommon.

## 1. Introduction

Over the past two decades, the incidence of renal cell carcinoma (RCC) has increased by about 2% annually, probably due to a greater detection of clinically silent localized RCC during imaging studies performed for other medical reasons and due to a growing incidence of risk factors all over the world, including a notably aging population, obesity and smoking [1].

Generally, in the case of incidental findings, renal lesions tend to be at an early-stage disease (T1a o T1b), and patients with T1 disease now make up more than half of all new patients with RCC.

This has led to the development of less invasive treatments compared to radical nephrectomy, such as nephron-sparing surgery (open, laparoscopic and robotic partial nephrectomy) or, more recently, percutaneous image-guided thermal ablations, aiming for competitive oncological outcomes together with a low incidence of operative complications [2].

Currently, percutaneous thermal ablation for renal cell carcinoma is considered an alternative treatment to partial nephrectomy, and it is suggested by numerous published guidelines [3,4].

Percutaneous thermal treatments are effective alternatives, especially for the management of fragile patients, such as the elderly, with many different comorbidities or solitary kidneys.

In this context, taking into account the greater risk of chronic kidney disease, percutaneous ablations could be considered an alternative minimally invasive treatment [5,6,7]. In these patients, the treatment goal should be local tumor control, but also a good preservation of renal function and a low complication rate with a relatively good quality of life. The EAU guidelines on Renal Cell Carcinoma 2022 reported that a shorter average length of hospital stay was found with the percutaneous technique, and a systematic review including 82 articles reported complication rates ranging between 8 and 20%, with most complications being minor [3].

Percutaneous ablations can be performed with different imaging techniques and multiple energy sources. Ultrasound (US) represents the most widely used technique for guiding ablations in the abdomen, particularly in Europe and Asia, while computed tomography (CT) is preferred in several centers, mainly in the United States [8], and MRI is promising, but not yet largely used.

Concerning the thermal agents, radiofrequency (RF) and microwave (MW) are heat-based methods, while cryoablation (CA) is the only method that uses a cold temperature to ablate the malignant tissues. Radiofrequency ablation was the first modality used, and long-term results are now available; cryoablation is considered comparable to RF in terms of outcomes, while MW has been introduced more recently, but the short amount of time needed for the ablations and their good efficacy are causing the use of this technique to grow quickly [9,10].

Even if these minimally invasive treatments represent available options for treating small RCCs, the choice of the thermal source is not yet codified and often depends on the equipment availability and operator experience [11].

Every thermal source has its procedural advantages and limitations, but what should matter in the end are the outcomes in terms of efficacy and safety. The purpose of our study was to retrospectively compare the radiological and clinical outcomes of percutaneous thermal ablations with radiofrequency and cryoablation guidance.

To perform such a comparison, in this paper, we analyzed the primary efficacy and recurrence rate as parameters to assess the effectiveness and used the complication rate and the pre- and post-procedural difference in the serum creatinine levels as parameters to assess the safety.

## 2. Materials and Methods

We retrospectively identified 82 adult patients (59 male and 23 female) with 90 T1 renal masses treated with imaging-guided percutaneous thermal ablations between January 2015 and December 2020.

Approval of the local ethic committees was obtained, and patients’ informed consent was waived.

### 2.1. Study Population

A total of 80 T1a tumors and 10 T1b tumors treated with percutaneous thermal ablation were included. A total of 43 tumors in 38 patients were treated with RFA (mean ± SD tumor size, 2.3 ± 0.9 cm), and 47 tumors in 44 patients were treated with cryoablation (mean ± SD tumor size, 2.1 ± 0.8 cm). Mean follow-up observation period was 26 ± 19 months. Major complications and efficacy, as measured using technical success and local tumor recurrence rates, were recorded.

Patients were treated in two different Northern Italian university hospitals: AOU of Parma in Parma (PR) and San Bassiano Hospital in Bassano del Grappa (VI).

Median age was 69.6 years old (IQR, 57–77; range, 31–88 years).

Adult patients with a renal tumor at T1a or T1b stage without any age limit were included.

The pre-treatment assessment of renal malignancy was proven via percutaneous core biopsy in 58 renal masses or based on CT and MRI findings using standard criteria for solid lesions, including contrast enhancement [12] and increasing diameter (>0.8 cm/year) during active surveillance [13,14], which cause suspicion for aggressive behavior.

According to the guidelines of the European Association of Urology [3], the American Urological Association [4] and the European Society Medical Oncology [15], we also included patients with comorbidities, renal failure, previous RCC, past nephrectomy, congenital malformations, solitary kidney and hereditary RCC.

Patients who had sepsis, severe coagulopathy, imaging signs of Gerota’s fascia or vessels invasion and the involvement of adjacent organs and lymph nodes (N > 0, M > 0 of TNM classification) were excluded.

### 2.2. Procedures

Indication of thermal ablation was given by the multidisciplinary board of the two hospitals, including interventional radiologists and urologists, on a case-by-case basis based on available devices and imaging techniques at the time of the treatment and operator preference.

Tumors were treated consecutively in each center with two different thermal ablative percutaneous methods: radiofrequency (*n* = 43) and cryoablation (*n* = 47). CT guide was used for the procedure in all ablations, and in 14 cases, a combined approach of US-CT was chosen.

The choice between radiofrequency ablation (RFA) and cryoablation depended on the centers’ expertise. One center used RFA exclusively, while the other used cryoablation, driven by their proficiency and available technology.

### 2.3. Ablative Devices and Techniques

All procedures were performed under local anesthesia and conscious sedation, with the patient lying in the most favorable position for direct needle approach. Adjunct procedures such as hydrodissection or pyeloperfusion were used upon team clinical judgment.

A percutaneous biopsy was performed, when feasible, before every procedure.

All RF procedures were performed using a monopolar system with a generation power of 250 W (RITA Medical System) linked with a 4-tined expandable electrode (StarBurst Talon) cooled with pumped saline solution (IntelliFlow Pump); target temperature in tissues to treat was about 105 °C.

The needle was 25 cm long and could cause variable extension of necrosis. Saline solution was dropped in tissues via the 4 expandable electrode tines to reduce tissue impedance and carbonization and to increase necrosis volume.

Cryoablations were performed with visual ice cryoablation. Cryoprobes were advanced percutaneously to just beyond the deep margin of the tumor. The number of cryoprobes used depended on the size of the lesion to ensure adequate ice ball coverage. A double-freeze cycle, with intervening active thaw, was applied. CT images were repeated during the freeze cycles and at the end of the procedure to confirm the formation of an adequate ice ball and the absence of complications. Initial freeze cycle was performed for 10 min at temperatures below −40 °C, and after at least an 8 min active thaw period, cryoablation was repeated for an additional 10 min and, finally, after at least 3 min of active thaw, the cryoprobes were removed. All patients were treated with Galil Medical 1.5 mm cryoprobes (17-gauge).

### 2.4. Imaging Guide

Unenhanced and contrast-enhanced CT scans were performed before the treatment to study tumor features (size, position, connection to adjacent organs, volume and contrast enhancement) and to confirm indication for treatment. The CT exam consisted of unenhanced scans (followed by corticomedullary and/or nephrographic phase when needed to better visualize the lesion and the vessels, especially in the case of endophytic tumors).

CT and multi-planar reconstruction images were used by the interventional radiologist to plan the point of entrance and to monitor the trajectory of the probe.

When the tumor was posterior and exophytic, US guide was used to define the site of entrance for the probe, its angle and the distance of the renal lesion from the skin, and the probe was inserted under live US guide; then, NECT or CECT allowed for the assessment of correct positioning and to perform any repositioning needed.

In some selected cases, the navigation system CAS-One^®^ IR (Cascination, Bern, Switzerland) was used to place the RF probe as desired.

Unenhanced and contrast-enhanced CT scans were performed at the end of the procedure to detect residual disease and to rule out perioperative complications.

In every patient treated with cryoablation, a CEUS was performed 24 h after the procedure.

### 2.5. Follow-Up

Follow-up included imaging examination at 1, 3, 6 and 12 months and every year thereafter, with the aim of detecting residual or recurrent disease and mid- to long-term complications. Different imaging modalities were used depending on the institution, including CECT, CEUS and MRI. CT follow-up included NECT and contrast-enhanced CT with arterial phase, portal venous phase and excretory phase.

MR included an axial T2-weighted, an axial T2-weighted fat-sat, axial and coronal dual-echo, axial dynamic 3D gradient-recalled echo before and after gadolinium injection (20, 70 and 180 s delayed) and 5 min delayed spoiled gradient-recalled echo imaging.

CEUS included a conventional grayscale examination and then a contrast-enhanced US with a bolus dose of prepared contrast agent (SonoVue, Bracco, Italy) injected into the cubital vein, followed by a rapid flushing with 5 mL of saline. Preferentially, the operators chose the same imaging modalities as the previous patients’ exams and maintained it during the months and the years after the procedure in order to promptly detect the recurrence.

### 2.6. Indicators of Efficacy

Ablation was considered successful if a complete tumor necrosis was obtained.

Tumor enhancement pattern and tumor size measurements on follow-up CT, MR or CEUS were investigated to assess residual or recurrent disease, as new areas of enhancement have been shown to be correlated with active tumor at pathologic analysis [16].

Primary treatment efficacy was defined as a technical successful ablation without any residual tumor in the first three months. Tumor was considered residual if lesion showed more than 10 HU enhancement on CT or signal enhancement on MRI and without the normal appearance of successfully ablated lesion (hyperintense compared with normal kidney parenchyma on T1 and hypointense to normal kidney parenchyma on T2 [17]) or persistent blood flow on CEUS at 0, 1 or 3 months.

Tumor recurrence was defined as a new enhancement within the ablated lesion during the follow-up by the sixth month of a previously documented successful treatment. Recurrence rate was defined as the percentage of the recurrent diseases.

Secondary efficacy was not considered as not all the residual or recurrent diseases were re-treated, and the number of the low rate of events might have determined an impossibility of detecting some statistically significant correlations. Overall survival was not considered because of the usually slow progression and low biological aggressiveness of treated T1 kidney tumors.

All such definitions were based on Cardiovascular and Interventional Radiological Society of Europe (CIRSE) guidelines [18].

Radiological reports of the follow-up exams served as reference standards for primary efficacy and presence of residual or recurrent disease.

### 2.7. Indicators of Safety

Complications were assessed using the revised Clavien–Dindo classification, which grades complications based on deviation from the normal postoperative course and level of intervention required. Any grade 3 or greater complication was considered a major complication.

Serum creatinine levels were recorded before and after the ablation procedures and the difference between these values was calculated.

Complications and serum creatinine levels were recorded retrospectively after the medical records were checked.

## 3. Statistical Analysis

The data analyses were performed using the statistical packages Jamovi 2.3 version (https://www.jamovi.org/; accessed on 14 February 2023) and IBM-SPSS v. 28. For the descriptive analysis of continuous variables, the main position, dispersion and shape indices were calculated, including the mean, median, fashion, variance, standard deviation, interquartile, minimum, maximum and range. Where it was relevant, the standard errors and their 95% confidence intervals were also reported. The qualitative characters, i.e., categorical data, were reported in frequency tables and expressed as absolute frequencies, relative frequencies and percentages.

Both parametric tests (Student’s *t*-test, ANOVA test and ANCOVA test) and non-parametric tests (Mann–Whitney test and Kruskal–Wallis test) were used for comparisons between the groups of continuous variables. Comparisons between the categorical variables were made using the chi-squared test and Fisher’s exact test.

For both continuous and categorical changeable variables, the results were considered statistically significant for a *p*-value of less than 5% (*p* < 0.05).

## 4. Results

The 43 tumors treated with RFA were obtained from 38 patients with a mean age of 69.5 years at the treatment (SD, 14.4 years; median, 75 years; range, 31–85 years), including 27 (71%) men and 11 (29%) women. A total of 12 (32%) patients had been previously treated for renal cell carcinoma (RCC), either in the same kidney or in the contralateral kidney; the remaining 26 (68%) patients had no history of renal cancer. All tumors underwent a single treatment session. The 47 tumors treated with cryoablation were obtained from 44 patients with a mean age of 69.8 years at the treatment (SD, 10.9 years; median, 70 years; range, 39–87 years), including 32 (72%) men and 12 (28%) women. A total of 8 (18%) patients had been previously treated for RCC, and the remaining 36 (82%) patients did not have a history of renal cancer. As with RFA, all tumors were treated during a single treatment session. There was not a statistically significant difference in the age (*p* = 0.91) or sex (*p* = 0.86) between patients who were treated with RFA and patients who were treated with cryoablation. Notably, 10 patients had multiple tumors that were treated either on the same date or on different dates. A comparison of the features studied and the technical outcome between the tumors treated with RFA and those treated with cryoablation is shown in Table 1. The mean tumor size for the patients treated with RFA was larger than that for patients treated with cryoablation (2.3 ± 0.9 vs. 2.1 ± 0.8 cm; *p* = 0.33). There were 12/43 centrally located masses among those treated with RF (28%), and 26/47 among those treated with cryoablation (55%)—*p* = 0.008.

The mean follow-up observation period was 29 months (range of 3.6–84 months) for the RF group and 25 (range of 5.4–62 months) for the CA group without a statically significant difference (*p* = 0.40).

### 4.1. Outcomes

There were four technical failures observed (95.6% technical success rate), three (6.9%) among the tumors treated with RFA and one (2.1%) among the tumors treated with cryoablation (*p* = 0.30).

**Figure 1:**
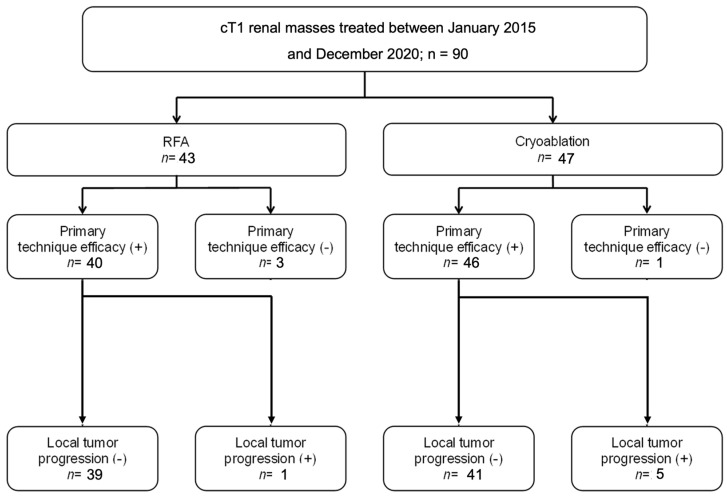
Number of effective ablations and local tumor progression in radiofrequency (RFA) and cryoablation (CA).

The overall local tumor recurrence was assessed for tumors that were followed for at least 3 months. Among the 40 tumors treated with RFA, after the exclusion of technical failures, 1 tumor (2.5%) showed local recurrence at 2.8 years after the procedure. Among the 46 tumors treated with cryoablation, 5 (10.8%) recurred locally at a mean of 0.9 years after the procedure (median, 1.0 years; range, 0.3–1.6 years)—*p* = 0.12. The characteristics of tumors that showed residual or recurrent disease are reported in Table 2. The results are summarized in Figure 1.

#### 4.1.1. Based on Dimensions

Regarding the 80 T1a (mean (±SD) tumor size, 2.2 ± 0.8 cm), the procedures resulted in 2/37 technically unsuccessful cases with RFA (5.4%) and 1/43 technically unsuccessful cases with CRYO (2.3%)-(*p*= 0.46). There were 1/35 recurrent diseases with RFA (2.8%) and 5/42 with CA (11.9%)-(*p*= 0.14).

Regarding the 10 T1b lesions (mean (±SD) tumor size, 4.0 ± 0.7 cm), the procedures resulted in 1/6 technically unsuccessful cases with RFA and 0/4 with CRYO (*p* = 0.90). No recurrence disease was detected in the T1b lesions.

#### 4.1.2. Based on Location

There were 52 exophytic lesions; 31 were treated with RFA, and 21 were treated with cryoablation. There were 3/31 technically unsuccessful cases and 1/28 recurrent disease case among those threated with RFA. No residual disease (*p* = 0.45) and 1/21 recurrent disease (*p* = 0.83) was detected among those threated with cryoablation.

There were 38 endophytic lesions; 12 were treated with RFA and 26 were treated with cryoablation (*p* = 0.008). No residual or recurrent disease was detected among those threated with RFA; there was 1/26 that was technically unsuccessful (*p* = 0.64) and 4/25 recurrent disease cases (*p* = 0.52) among those threated with cryoablation. The results are summarized in Table 3.

### 4.2. Complications

Among the 43 RFA procedures performed, 1 (2.3%) resulted in a major complication, particularly a urinary fistula with stranding of contrast medium from the pelvis. The treated lesion was a 3.5 cm endophytic mass in an 85-year-old woman. Among the 47 cryoablation procedures, no major complications occurred. There was not a statistically significant difference in the occurrence of a major complication between the RFA and cryoablation procedures (*p* = 0.93).

Overall, 2 peri-renal hematomas (2/43) were detected after the RF procedure and 11/47 were detected after the cryoablation (*p* = 0.01).

The serum creatinine level difference before and after the procedure was unremarkable (range: −0.05 to 0.05 mg/dL), without a statistically significant difference between the two groups (*p* = 0.86).

## 5. Discussion

Although the comparisons between thermal ablation and partial nephrectomy have been evaluated, direct comparisons of the different thermal ablative modalities for therapeutic efficacy and clinical outcomes are still limited.

The present study aimed to provide a comprehensive analysis of the therapeutic efficacy and clinical outcomes of different thermal ablative modalities, specifically radiofrequency ablation (RFA) and cryoablation (CA), in the treatment of renal tumors. The primary efficacy rates observed in our series of 90 procedures were 93.1% for RFA and 97.9% for cryoablation, with tumor recurrence rates of 2.5% for RFA and 10.8% for cryoablation, during a mean follow-up time of 2.2 ± 1.6 years. In addition, the preservation of renal function as measured using the serum creatinine levels was observed in all treatment groups.

These results are consistent with previous studies that evaluated the effectiveness of percutaneous RF or CA in treating renal masses, demonstrating technical effectiveness rates ranging from 87% to 97% for small tumors measuring 4 cm or less [19,20,21,22].

Over recent years, a similar efficacy between RF and cryoablation was established by numerous published papers [10,23,24,25,26,27,28,29] and is now stated by the American Urological Association guidelines [4].

In particular, the AUA guidelines report that “it is generally accepted that oncologic outcomes are similar for both approaches”, while the ESMO guidelines report that RFA presents “slightly higher local recurrence rate compared with CA” [15].

In 2019, Zhou et al. [23] conducted a review that included 297 cases of biopsy-proven T1a RCCs treated with CT-guided RFA (82%), cryoablation (26.9%) and MWA (27.9%). The study found similar rates of technical success for all three treatments (*p* = 0.33). While RF ablation and MWA showed higher rates of primary efficacy than cryoablation at 1 month post-ablation, there were no significant differences in local recurrence after a follow-up period of 2 years.

Thompson et al. [30] investigated 1057 partial nephrectomy procedures (PN), 180 percutaneous RFA procedures and 187 percutaneous cryoablation procedures and reported the local recurrence-free survival rates at 3 years in cT1a tumors for PN, RFA and cryoablation of 98%, 98% and 98%, respectively. There was not a statistically significant difference in the local recurrence-free survival among the three treatments (*p* = 0.49) for all patients.

Our findings align with the results of these studies. We did not observe statistically significant differences in the primary efficacy and tumor recurrence rates between the RF and CA treatments for T1 lesions over a medium follow-up time of 2.2 ± 1.6 years (*p* = 0.30 and *p* = 0.12, respectively).

**Figure 2:**
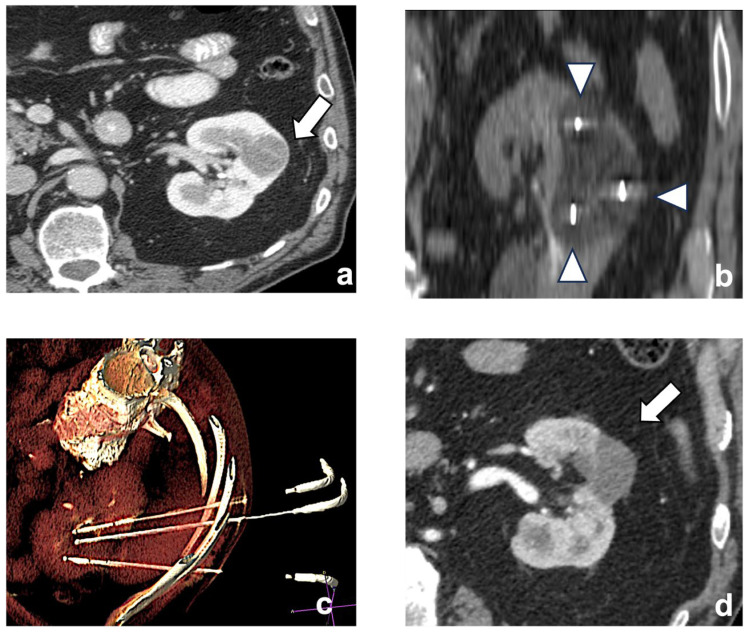
A left chromophobe RCC (arrow), hypovascular compared to normal renal cortex (**a**). Three cryoablation probes (arrowheads) are placed in the tumor, creating an ice-ball ((**b**) coronal plane). A volume-rendering reconstruction represents the probes’ access and positioning in the renal mass (**c**). Post-contrast CT shows the positive outcome of the ablation (arrow (**d**)).

Historically, size is a well-known factor of technical unsuccess and tumor recurrence. This is particularly true when the ablative treatment is performed with RF. On the other hand, CA allows for the extension of the ablation area using multiple cryoprobes, generating ice balls that are large enough to treat tumors as large as 8 cm in size [31] (Figure 2).

At the state of the art, the current guidelines only recommend thermal ablations for smaller tumors (notably, T1a) [18].

Another crucial factor to consider is the tumor location, as centrally located tumors are more difficult to treat given their proximity to hilar structures. In particular, location has been linked with different outcomes related to the energy source, as it is known that RFA suffers of “heat sink” effects originating in the highly vascular renal hilum [32], thus limiting the treatment of centrally located tumors. However, even in CA, the “cold-sink effect” is reported, which could affect the treatment efficacy when high-flow vascular structures are in the vicinity of the tumor [33] by raising the temperature of the ice ball.

In our study, the analysis of subgroups based on dimensions (T1a vs. T1b) and location (endophytic vs. exophytic) did not show any significant difference between RFA and CA. This could probably be related to the low numerosity of T1b lesions (*n* = 10) and of centrally located tumors treated with RFA (*n* = 12) that might have influenced the statistical power; moreover, centrally located tumors were treated significantly more often with CA, generating a tumor selection bias. However, also other studies have previously reported no differences between RFA and CA concerning dimensions or location.

**Figure 3:**
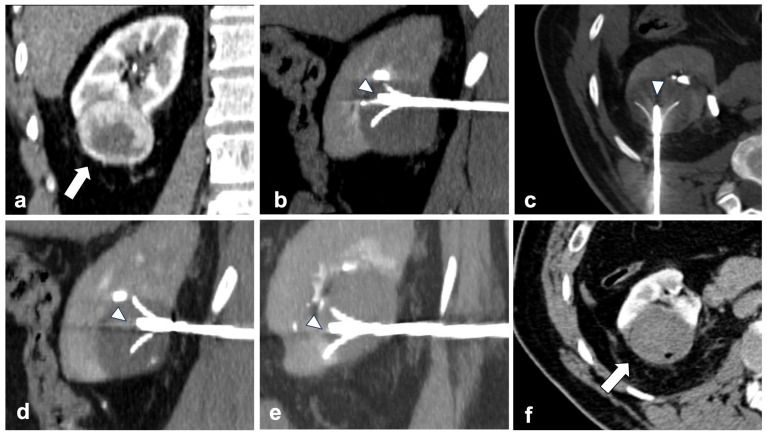
RFA is possible and effective even in T1b lesions. (**a**) shows a right lower pole exophytic clear cell RCC (arrow—coronal plane) measuring 52 × 44 mm. A 4-tined expandable electrode (arrowheads) is positioned into the upper-front part of the mass ((**b**) sagittal plane). Then, two other positionings are made by receding the probe (arrowhead) backward ((**c**) axial plane and (**d**) sagittal plane)) and repositioning the needle (arrowhead) in the lower portion of the mass ((**e**) sagittal plane). One month after ablation, the mass shows no enhancement (arrow), meaning tumor necrosis is achieved ((**f**)—axial plane).

In relation to dimensions, in 2018, Hasegawa et al. [27] conducted a direct comparison on the clinical outcomes between 23 radiofrequency ablation procedures (RFA) and 23 cryoablation procedures for the treatment of clinical T1b renal cell carcinoma. They reported that there was no significant difference between the local tumor progression rate after RFA and cryoablation (*p* = 0.66). Indeed, RF allows for multiple overlapping ablation in a single treatment session, allowing for lesions bigger than the expandable needles’ ablation area to be covered (Figure 3).

Concerning the tumor location, Atwell et al., in a series of 445 thermal ablations, examined the estimated local recurrence-free survival rates at 1, 3 and 5 years for central tumors treated with RFA and cryoablation, and did not find a statistically significant difference (*p* = 0.08) [31]. Particularly, the use of ancillary techniques to minimize the risk of complications, such as double-J ureteral stenting or retrograde pyeloperfusion, grants the operator with the ability to be more radical in the ablation, thus reaching the same outcomes of cryoablation.

The overall complication rate (10%) in the present series is comparable to that reported in a large series of renal percutaneous ablations in the literature [17,33,34,35].

The major complication rate (Grade III or higher) was 2.3% in the RF group and 0% in the CA group, and no statistically significant difference was found in the occurrence of major complications between the two groups (*p* = 0.93).

CA showed a higher incidence of peri-renal hematomas (Grade I, without the need for further interventions) (*p* = 0.01) without any relations to tumor locations. Indeed, CA is associated with a higher risk of hemorrhage because the surrounding blood arteries are not directly cauterized, such as in RFA, where heat has an intrinsic coagulative effect and reduces the risk of minor hemorrhage. Moreover, CA usually requires the insertion of multiple probes to create an adequate ice ball, and the procedure is longer, resulting in a higher amount of tissue damage [36].

Finally, the major complication was a urinary fistula with a stranding of contrast medium from the pelvis that occurred during an RFA of a 3.5 cm endophytic mass in an 85-year-old woman that required an endoscopic insertion of a double-J catheter and prolonged hospitalization (20 days). Even if the heat sink effect could be a protective factor for endophytic tumors treated with RF, heating the renal hilum has shown to be more dangerous than cryoablations in terms of the damage caused to central structures (vessels and the urinary tract).

The serum creatinine levels before and after the procedure did not show a significant difference (*p* = 0.86), thus confirming that percutaneous ablations are safe procedures compared to PN, regardless of the energy source.

There are several limitations to our study. The main ones include the retrospective study nature and the limited number of patients.

The criterion for enrolling tumors into ablations included not only biopsy-proven RCC, but it was also based on imaging features and was therefore subject to errors, as up to 15–20% of small (<4 cm) masses that undergo resection are benign [37], thus limiting the accuracy of the tumor local progression rate [38,39]. However, in some cases, the imaging characteristics of the tumors were highly consistent with renal cell carcinoma, and the clinical team had a high degree of confidence in the diagnosis based on these findings (lesion with prevalent enhancement in the nephrographic and venous phase with the presence of small central calcifications). In some cases, they were recurrences or residuals of renal tumors that were previously operated on (*n* = 25) and histologically characterized, and in sporadic cases, they were patients affected by syndromes (Von Hipple Lindau—*n* = 3), which present a high incidence of renal tumors.

Another factor influencing the decision was the relatively high rate of non-diagnostic biopsies reported in the literature. In certain cases, with clear imaging evidence, it was preferred to avoid subjecting patients to a procedure that might yield inconclusive results, potentially leading to delays in treatment without providing additional actionable information; in such instances, the decision was made not to perform an RMB because it was deemed unlikely to significantly alter the treatment plan or outcome.

While the EAU guidelines suggest that the best option is to perform the biopsy and the ablative treatment in two separate sessions, it is important to consider the context of the patient population. Many of the patients were elderly and from distant locations, which posed challenges for multiple procedural sessions. In these cases, a more streamlined approach was preferred to minimize patient discomfort and logistical burdens.

We acknowledge that during thermal ablation, a track ablation is commonly performed at the end of the procedure. However, conducting a biopsy in a separate session introduces an inherent risk of seeding tumoral cells along a biopsy needle path that is not going to be exactly the same as the ablation needle, which could lead to potential complications or the spread of cancer cells along the biopsy tract.

The EAU guidelines still recommend performing a biopsy prior to the treatment in the same session. In some instances (*n* = 14), the procedures were conducted under both CT and ultrasound guidance. Performing the biopsy before TA under ultrasound guidance could potentially alter the visualization of the lesion if both the biopsy and treatment are carried out within the same session. This concern stems from the potential visual disruption caused by the biopsy procedure, impacting the lesion’s clarity during the subsequent needle insertion under US, thus making it necessary to perform the procedure under the CT guide.

Another limitation is the lack of patient characteristics, like the Charlson Cormobidity Index and the stages of Chronic Kidney Disease, or the lack of tumor classifications according to the ABLATE or R.E.N.A.L. nephrometry score.

As opposed to the limitations, the strength of this study is that it includes the experience of two different institutions that use two different energy sources for the percutaneous ablation and where operators have achieved high experience on the energy source used, allowing for the generalization of our results.

## 6. Conclusions

In conclusion, the results of the present study show that the thermal ablation of T1 renal masses with radiofrequency ablation and cryoablation is associated with high technical success and local tumor control, even when considering only one treatment setting, and suggest that both RF ablation and cryoablation are safe and effective.

Prospective randomized intermediate and long-term trials are necessary to establish an objective basis for ablative modality selection for the treatment of T1 RCC.

## Figures and Tables

**Table 1:** Comparison of features and technical outcomes between tumors treated with RFA and CA.

	RFA	Cryoablation	*p*
Number of tumors	43	47	
Number of patients	38	44	
Mean age at treatment (years)	69.5	69.8	0.91
Male patients no. (%)	27 (71)	32 (72)	0.86
Prior RCC treatment n° (%)	12 (32)	8 (18)	
Single treatment session (%)	100	100	
Mean tumor size (cm)	2.3 ± 0.9	2.1 ± 0.8	0.33
Centrally located tumors n° (%)	12 (28)	26 (55)	0.008
Mean follow-up (months)	29	25	0.40
T1b tumors n° (%)	6/43 (13.9)	4/47 (8.5)	0.41
Technical failure rate n° (%)	3/43 (6.9)	1/47 (2.1)	0.30
Local tumor recurrence rate n° (%)	1/40 (2.5)	5/46 (10.8)	0.12
Major complications rate n° (%)	1/43 (2.3)	0/47 (0)	0.93
Peri-renal hematoma rate n° (%)	2 (4.6)	11 (23.4)	0.01

**Table 2:** Characteristics of tumors that showed residual or recurrent disease.

	Residual/Recurrent	Location	Size (cm)	Solitary Kidney	Histology
RFA	Residual	Exophytic	5.6	Yes	Collecting Duct Ca
	Residual	Exophytic	1.7	Yes	Chromophobe
	Residual	Exophytic	1.8	Yes	Chromophobe
	Recurrent	Exophytic	2.8	No	None
CA	Residual	Endophytic	3.0	No	Clear Cell
	Recurrent	Exophytic	2.0	No	Clear Cell
	Recurrent	Endophytic	1.0	No	Clear Cell
	Recurrent	Endophytic	1.6	No	Papillary
	Recurrent	Endophytic	1.8	No	Clear Cell
	Recurrent	Endophytic	2.2	No	Chromophobe

**Table 3:** Technical outcomes and recurrence rates based on tumor dimensions and location.

Outcomes	T1a (*n* = 80)	T1b (*n* = 10)	Exophytic (*n* = 52)	Endophytic (*n* = 38)
Technical success rate n° (%) with RFA	35/37 (94.6)	5/6 (83.3)	28/31 (90.3)	12/12 (100)
Technical success rate n° (%) with CA	42/43 (97.7)	4/4 (100)	21/21 (100)	25/26 (96)
Recurrent disease rate n° (%) with RFA	1/35 (2.8)	0/5 (0)	1/28 (3.6)	0/12 (0)
Recurrent disease rate n° (%) with CA	5/42 (11.9)	0/4 (0)	1/21 (4.8)	4/25 (16)

## Data Availability

The data are available upon request to the corresponding authors.
